# Commentary: A Randomized, Double-Blind, Placebo-Controlled Trial of Metformin Treatment of Weight Gain Associated with Initiation of Atypical Antipsychotic Therapy in Children and Adolescents

**DOI:** 10.3389/fpsyt.2017.00059

**Published:** 2017-04-19

**Authors:** Jeffrey Samuel Goltz, Timothy Reynolds Rice

**Affiliations:** ^1^Psychiatry, Mount Sinai St. Luke’s-West Hospital, New York City, NY, USA; ^2^Psychiatry, Icahn School of Medicine at Mount Sinai, New York City, NY, USA

**Keywords:** metformin, atypical antipsychotics, children and adolescents, obesity, weight gain

## Introduction

In 2006, Klein and colleagues conducted the first randomized control trial (RCT) to analyze the benefit of adding metformin to treat weight gain induced by second-generation antipsychotics (SGAs) in children and adolescents (1). In this commentary, I review this study, the relationship between childhood obesity and SGA weight gain, and current methods for treating SGA-induced weight gain to better elucidate this trial’s significance.

## Introduction to Childhood Obesity

Youth obesity has reached epidemic proportions around the world. Obesity impacts both youth physical and mental health. Overweight habitus creates multiple physical limitations, increases the incidence of multiple medical comorbidities including diabetes and hypertension, and raises the rates of morbidity and mortality at adulthood (2, 3). In addition, obesity has been associated with increased economic costs and various impairments in mental health, including a decrease in self-esteem (4), ADHD, depression (4, 5), eating disorders (4), and increased rates of stigmatization (2).

## Weight Gain Induced by SGAs

Second-generation antipsychotics are used for the treatment of mania, psychosis, depression, impulsivity, and aggression in children and adolescents (6). While weight gain in all age groups is a common adverse drug effect, youth are more vulnerable to this effect relative to adults (7). For example, aripiprazole, considered by some to be weight neutral in adults, causes significant weight gain in children and adolescents (7). SGA-induced weight gain contributes to non-compliance with medication (8). For these and other reasons, behavioral and medicinal means to reduce the extent of weight gain are under active investigation.

## Evidence for Lifestyle Interventions for Weight Loss

The positive effects of lifestyle interventions on reducing SGA-induced weight gain and on overall mental health make them an attractive treatment option. A brief description of some of the evidence for the efficacy of lifestyle interventions will be provided. A recent meta-analysis of adults has indirectly demonstrated that the amount of weight loss due to lifestyle interventions may be similar to that provided by use of pharmaceutical agents (9). Though contradictory findings exist, including a study (10) in which medication alone was more effective for weight loss than behavioral interventions, it is possible that this study reflects the difficulty of many psychiatric patients to adhere to strict dietary guidelines or exercise regimens, including those used in behavioral interventions (10, 11). This is one reason that medications like metformin are being studied.

## Metformin as a Medication for Weight Loss

Metformin is a biguanide antidiabetic agent. Multiple mechanisms have been proposed for its effect on weight loss. These include its ability to decrease insulin resistance (reversing the insulin resistance caused by antipsychotics) and reduce hepatic glucose production (11, 12). While multiple RCTs and meta-analyses show metformin to be moderately beneficial for promoting weight loss in adults using antipsychotics, others found no weight loss benefits (13–15). The weight-reducing potential of metformin in non-diabetic, overweight children, and adolescents has been previously demonstrated (16). Metformin’s efficacy in treating antipsychotic-induced weight gain in youth has been examined in open label trials and case reports (14, 17). However, only three RCTs examining its efficacy in treating antipsychotic-induced weight gain in children have been published (1, 11, 18).

## Klein’s RCT

Klein et al. (1) was the first RCT to examine metformin’s weight-reducing potential in children receiving antipsychotic medication. Thirty-eight children with varying psychiatric diagnoses aged 10–17 on one SGA for less than 12 months (olanzapine, risperidone, or quetiapine) and exhibiting weight gains greater than 10% of their predrug weight were randomized to receive either metformin (850 mg BID after titration) or placebo. A dietician assessed all subjects and counseled them in improving their diet and exercise regimens. Primary outcome measures were changes in weight and body mass index (BMI) over a 16-week period. The metformin-treated group showed minimal weight (mean = −0.13 kg, SD = 2.88) and BMI (mean = −0.43, SD = 1.07) changes, compared to those in the placebo group who gained on average 4.01 kg (SD = 6.23) and mean increases of 1.12 (SD = 2.02) in BMI. When correcting for expected weight gain in developing children, the final *z*-score was 1.72 (SD = 0.99) for the placebo group’s weight change and 0.09 (SD = 0.25) for BMI, versus *z*-score of −0.14 (SD = 0.29) and −0.14 (SD = 0.20), respectively, in the metformin-treated group. These results were statistically significant. Also the rate of children with an indication for a glucose tolerance test was greater in the placebo group (*p* < 0.01), and consequently, more children taking placebo had impaired glucose tolerance. There was no significant difference in rates of adverse events.

## Subsequent Studies

Anagnostou et al. (11) subsequently examined the impact of metformin on weight reduction in 61 children (ages 6–17) with autism spectrum disorder taking various SGAs. This study found that metformin was more effective than placebo in treating antipsychotic-induced weight gain. In contrast, Arman et al. (18) failed to establish the efficacy of using metformin to prevent weight gain in a study of 32 children/adolescents with schizophrenia or schizoaffective disorder receiving both risperidone and metformin versus placebo in a 12-week RCT trial.

## Conclusion

Thus, data from existing RCT-based studies for evaluating the benefits of using metformin to reduce antipsychotic-induced weight gain in youth have been contradictory. Existing data have limitations, including small sample size, inconsistent cross-study methodologies, and limited time frames. Larger and more longitudinal RCTs are needed and are currently underway [see IMPACT trial by Reeves et al. (19)] and will likely shed more light on the efficacy of using metformin for treatment and prevention of SGA-induced weight gain in youth.

## Author Contributions

JG—substantial contribution to designing the work, acquisition of information, drafted and revised the work, approved final version, and agreed to be accountable for all aspects of the work. TR—substantial contribution to designing and outlining the work, revised it for important intellectual content, approved final version, and agreed to be accountable for all aspects of the work.

## Conflict of Interest Statement

The authors declare that the research was conducted in the absence of any commercial or financial relationships that could be construed as a potential conflict of interest. The reviewer, GR, and handling editor declared their shared affiliation, and the handling editor states that the process nevertheless met the standards of a fair and objective review.
